# Exploring the Accuracy Limits of PNO-Based Local Coupled-Cluster Calculations for Transition-Metal Complexes

**DOI:** 10.1021/acs.jctc.3c00087

**Published:** 2023-03-14

**Authors:** Ahmet Altun, Christoph Riplinger, Frank Neese, Giovanni Bistoni

**Affiliations:** † Max-Planck-Institut für Kohlenforschung, Kaiser-Wilhelm-Platz 1, D-45470 Mülheim an der Ruhr, Germany; ‡ FAccTs GmbH, Rolandstrasse 67, 50677 Köln, Germany; § Department of Chemistry, Biology, and Biotechnology, University of Perugia, 06123 Perugia, Italy

## Abstract

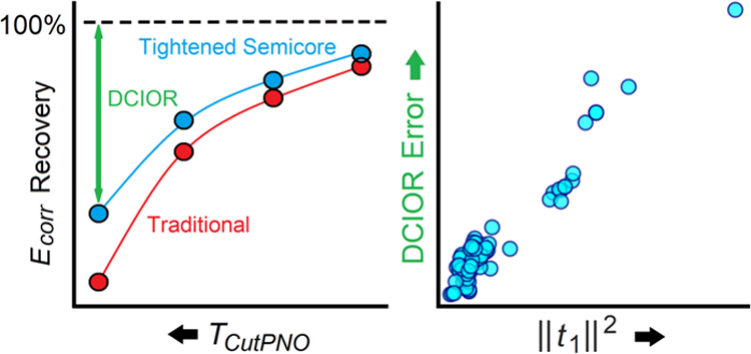

While the domain-based local pair natural orbital coupled-cluster method with singles, doubles, and perturbative triples (DLPNO-CCSD(T)) has proven instrumental for computing energies and properties of large and complex systems accurately, calculations on first-row transition metals with a complex electronic structure remain challenging. In this work, we identify and address the two main error sources that influence the DLPNO-CCSD(T) accuracy in this context, namely, (i) correlation effects from the 3s and 3p semicore orbitals and (ii) dynamic correlation-induced orbital relaxation (DCIOR) effects that are not described by the local MP2 guess. We present a computational strategy that allows us to completely eliminate the DLPNO error associated with semicore correlation effects, while increasing, at the same time, the efficiency of the method. As regards the DCIOR effects, we introduce a diagnostic for estimating the deviation between DLPNO-CCSD(T) and canonical CCSD(T) for systems with significant orbital relaxation.

## 1 Introduction

Recent advances in exploiting the local nature of electron correlation have enabled the development of linear scaling variants of the “gold standard” CCSD(T) method^1^ of quantum chemistry, *i.e*., the coupled-cluster method with singles, doubles, and perturbative triples. In particular, the domain-based local pair natural orbital CCSD(T) method [DLPNO-CCSD(T)]^2−11^ has proven particularly effective.^12−14^

In the DLPNO-CCSD(T) framework, the virtual space associated with each electron pair is spanned by a compact set of pair natural orbitals (PNOs),^2^ and only those with an occupation number greater than *T*_CutPNO_ are included in the correlation space. This truncation of the virtual space introduces an error in the DLPNO-CCSD(T) energy that can be reduced by tightening the *T*_CutPNO_ threshold. The error converges to zero at the *T*_CutPNO_ = 0 limit.

Extensive benchmark studies on reactions involving main group elements^15−20^ have shown that, when the so-called “TightPNO” settings (*T*_CutPNO_ = 10^–7^ and all other thresholds in the DLPNO machinery set to conservative values)^9,10^ are used, DLPNO-CCSD(T) typically retains 99.9% of the canonical CCSD(T) correlation energy, enabling chemical accuracy for most applications. However, for systems with a complex electronic structure, tighter *T*_CutPNO_ values might be necessary, which necessarily reduces the efficiency of the methodology. As an alternative to tightening the *T*_CutPNO_ threshold for approaching the complete PNO space (CPS) limit with a given atomic orbital basis set, extrapolation schemes that exploit the smooth dependence of the correlation energy on the *T*_CutPNO_ parameter can be used.^21,22^ In particular, the two-point CPS(6/7) extrapolation scheme^21,22^ allows DLPNO-CCSD(T) relative energy calculations with sub-kJ/mol accuracy with respect to canonical CCSD(T).^21−24^ It is worth mentioning here that other extrapolation approaches have been suggested for local correlation methods with varying degree of accuracy.^14,25−28^

Hence, for the chemistry of main group elements, local correlation approaches such as DLPNO-CCSD(T) have reached the accuracy needed for most intent and purposes. However, calculations involving transition metals (TMs) with a complex electronic structure remain challenging.^29−31^ For example, Jan Martin and co-workers pointed out a possible link between the error in the correlation energy of PNO-based local CCSD(T) methods and static correlation effects.^32,33^ In addition, subvalence correlation effects often play a role in TM chemistry,^34^ and the accurate inclusion of such effects might be challenging with local coupled-cluster schemes.^35^ The aim of this study is to elucidate the origin of such deviations and to propose computational strategies to deal with these shortcomings of local correlation methods. As a case study, we focus on the MOBH35 benchmark set^36,37^ of the first-, second-, and third-row closed-shell TM complexes. This set contains relative energies varying between −54 and +84 kcal/mol.

## 2 Computational Details

The coordinates of the complexes in the MOBH35 set were taken from refs (36) and (37). The canonical CCSD(T)/def2-SVP energies at these geometries^33^ were considered as benchmark data for the present DLPNO-CCSD(T)/TightPNO/def2-SVP results.

All calculations were performed in this study with a development version of the ORCA program package based on version 5.0.^38−41^ SCF and canonical CCSD(T) calculations were carried out without any resolution of the identity (RI) approximation. In contrast, the DLPNO-CCSD(T) method exploits the RI approximation, and thus, “/C” auxiliary bases are needed in these energy calculations. These were generated with the automated auxiliary basis set construction module of ORCA (i.e., “autoaux”) with maximum possible angular momentum.^42^

In the DLPNO-CCSD(T) calculations, the augmented Hessian Foster–Boys (AHFB) scheme was employed for localizing occupied orbitals.^43^ Unless otherwise specified, the perturbative triples correction was performed using the accurate iterative (*T*_1_) algorithm.^44,45^ The results with the semicanonical (*T*_0_) approximation^46^ are provided in the Supporting Information.

All DLPNO-CCSD(T) calculations were performed with TightPNO settings.^9,10^ The *T*_CutPNO_ parameter was set to 10^–*X*^ (*X* = 6, 7, 8, and 9). The resulting correlation energies were also extrapolated to the CPS limit, as detailed in refs (21) and (22).

For estimating the parameters of the dynamic correlation-induced orbital relaxation (DCIOR) contribution introduced in Section 3.2, regression analyses were performed on the DLPNO-CCSD(*T*_1_)/TightPNO/def2-SVP (*T*_CutPNO_ = 10^–*X*^) data using the L2-regularized Huber loss that minimizes both the mean absolute error (MAE) and mean square error (MSE) to some extent,^47^ as implemented in the Python’s scikit-learn library (complexity parameter α = 10^–4^; the hyper parameter controlling the number of samples to be classified as outliers ε = 1.35).^48^

To assess the basis set dependency of the DCIOR contribution, canonical and DLPNO-CCSD(T)/TightPNO calculations were performed on reaction 32 of the MOBH35 set in conjunction with Karlsruhe def2 (def2-SVP, def2-TZVP, and def2-QZVP)^49^ and Dunning correlation-consistent cc-pVnZ (n = D, T, and Q) and aug-cc-pVnZ (*n* = D and T) basis sets.^50−52^ Default ORCA settings were used in conjunction with def2-type basis sets: all-electron basis sets were used for the first-row TMs, while Stuttgart–Dresden effective core potentials (SD-ECPs) (see ref (53) and the references therein) were used for the second- and third-row TMs. When the Dunning basis sets were used, the (aug-)cc-pVnZ-PP basis set with a relativistic SK-MCDHF-RSC pseudopotential was assigned to the Pt atom.^54^

## 3 Results and Discussion

### 3.1 Subvalence Correlation Effects

Subvalence correlation effects play an important role in TM chemistry.^34,55−57^ For this reason, the ORCA^38−41^ program package uses very conservative frozen-core (FC) settings^35^ in correlated calculations: for each element, orbitals with energy lower than −200 eV are excluded from the correlation treatment and form the so-called “reduced” core (or chemical core), while those with energy higher than −80 eV are correlated. If the energy of an orbital falls between these regions, then some physically relevant assumptions, such as the consistency of the reduced core region for elements in the same group, are used in determining whether the orbital is correlated or not.^35^

An especially important class of orbitals that is excluded from the reduced core is the so-called 3s and 3p semicore orbitals of first-row transition metals, which are known to contribute to metal–ligand σ interactions in organometallic complexes.^34^ Previous calculations demonstrated that, while such semicore electrons are quite localized at the HF level, they relax significantly in CASSCF calculations by interacting with the electrons in the valence shell.^34^ In addition, electron correlation from such orbitals was found to be strongly dependent on the basis set and method of choice, and especially large errors could be found with perturbative approaches.^55−57^

**Figure 1:**
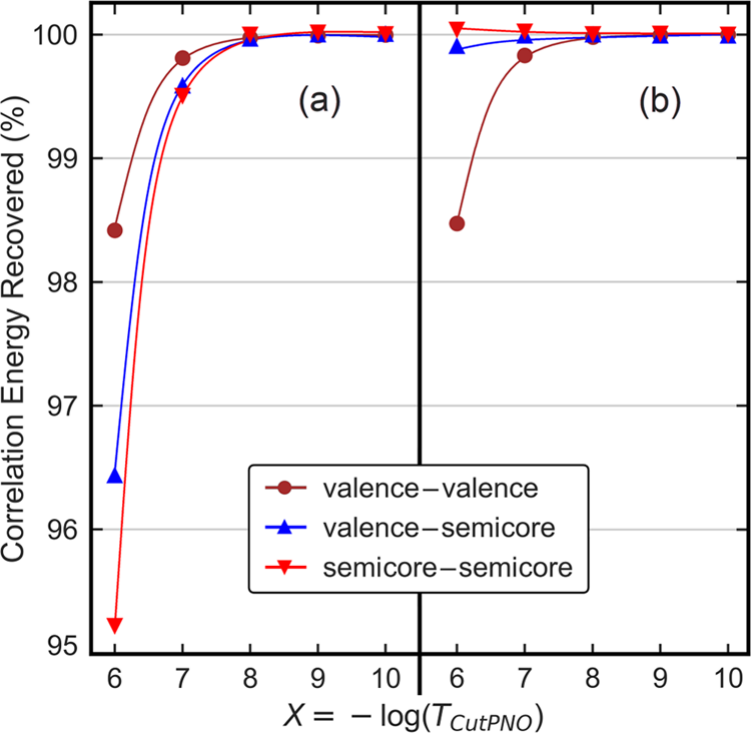
Dependence of the percent recovery of DLPNO-CCSD/TightPNO/cc-pwCVQZ strong-pair correlation energies of the Zn atom for valence–valence, valence–semicore, and semicore–semicore contributions on the size of the PNO space controlled by the *T*_CutPNO_ threshold set to 10^–*X*^ (*X* = 6–10) in reference to the results at the *T*_CutPNO_ = 0 limit (a) when the same *T*_CutPNO_ is used for all electron pairs and (b) when *T*_CutPNO_ is tightened 100 times for the valence–semicore and semicore–semicore pairs. All electron pairs were included in the correlation treatment, and occupied orbitals were not localized for obtaining a consistent set of electron pairs in all calculations.

Considering that the virtual space in DLPNO-CCSD(T) calculations is spanned by PNOs, which are obtained from the local MP2 (LMP2) amplitudes, we start our analysis by investigating the accuracy of the DLPNO-CCSD(T) scheme for the calculation of 3s3p semicore correlation energies. The percentage of semicore–semicore, semicore–valence, and valence–valence correlation energies recovered by DLPNO-CCSD with truncated PNO spaces in reference to DLPNO-CCSD pair correlation energies at the *T*_CutPNO_ = 0 limit is shown in Figure 1a (the Zn atom was considered as a case study).

All of the components of the correlation energy are well converged with *T*_CutPNO_ = 10^–8^, while for smaller *T*_CutPNO_ values, the semicore–semicore and semicore–valence components of the correlation energy show large deviations from the values at the *T*_CutPNO_ = 0 limit. These results are consistent with previous all-electron DLPNO-CCSD(T) calculations,^35^ showing that convergence toward the canonical limit is faster for electron pairs with both orbitals in the valence region. This effect had been attributed to the large energy separation between the electrons in the reduced core and the virtual orbitals, which causes the corresponding LMP2 amplitudes to vanish.^35^ Therefore, by default, ORCA uses more conservative *T*_CutPNO_ values for electron pairs involving core orbitals.

A similar strategy is tested here for the semicore orbitals, *i.e.*, a *T*_CutPNO_ value 100 times smaller is used for the pairs involving semicore electrons
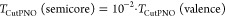
1

Hereafter, this scheme will be denoted as the “tightened semicore settings”, while the scheme that uses the same *T*_CutPNO_ value for all electron pairs that are correlated by default will be denoted as “traditional settings”. The results for the Zn atom using the tightened semicore settings (Figure 1b) demonstrate that the error in the semicore–semicore and valence–semicore correlation contributions vanishes with this strategy.

**Figure 2:**
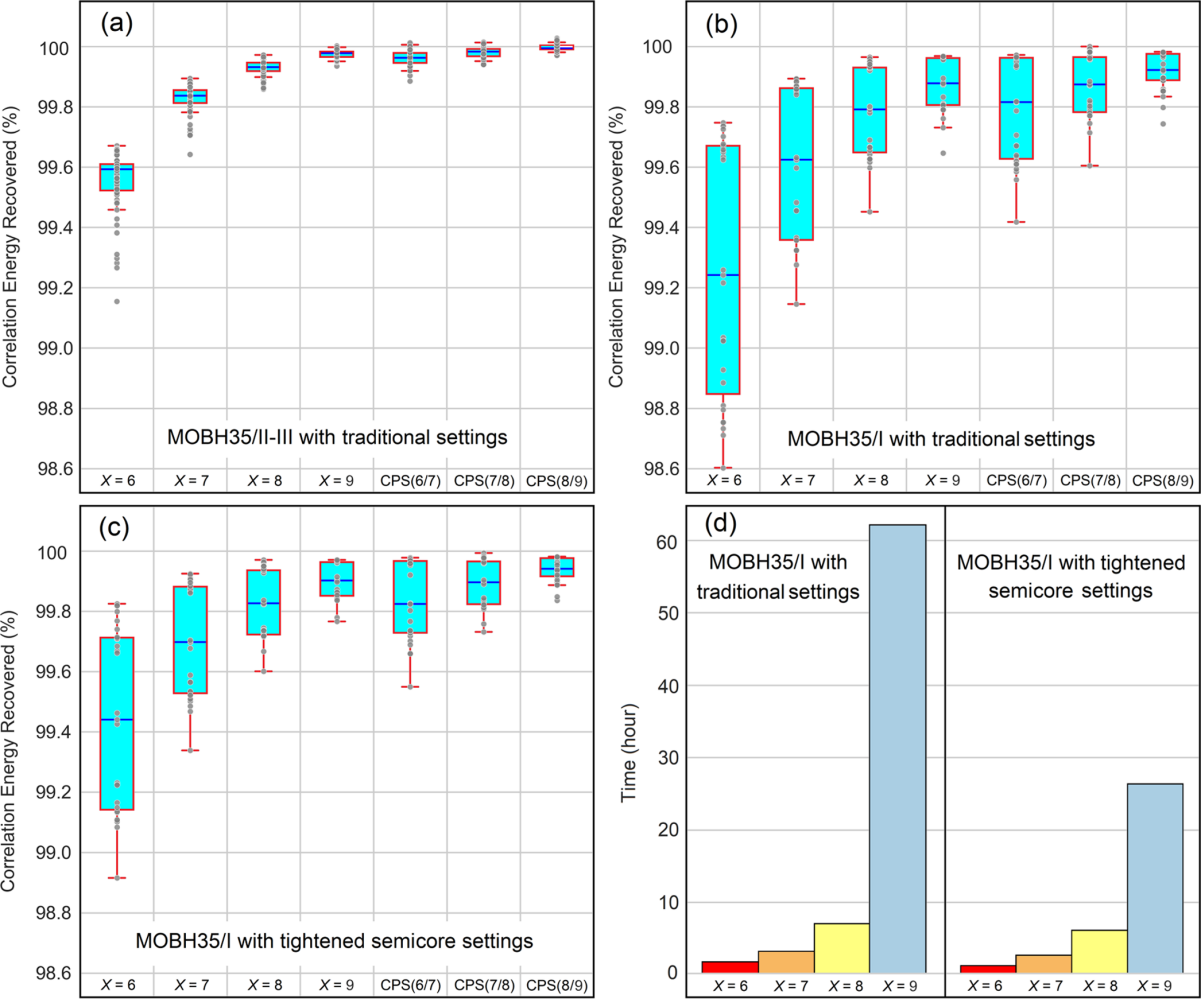
In reference to the CCSD(T)/def2-SVP correlation energies, the percent recovery of the DLPNO-CCSD(T)/TightPNO/def2-SVP absolute correlation energies with varying sizes of PNO spaces controlled by the *T*_CutPNO_ threshold set to 10^–*X*^ (*X* = 6–9) and with different CPS(*X*/*Y*) schemes on all complexes of (a) the MOBH35/II-III subset with the traditional settings, (b) the MOBH35/I subset with the traditional settings, and (c) the MOBH35/I subset with the tightened semicore settings. (d) Average computational time of the DLPNO-CCSD(T)/TightPNO/def2-SVP correlation energies of the complexes in the MOBH35/I subset with the traditional and tightened semicore settings. 16 cores from a single cluster node equipped with four Intel Xeon CPUs were used for all complexes.

To test whether this approach could help increasing the general accuracy of DLPNO-CCSD(T) for challenging TM complexes, we considered the MOBH35 benchmark set. To assess the role of the 3s3p correlation, this benchmark set was divided into two subsets, namely, MOBH35/I and MOBH35/II-III. The MOBH35/I contains reactions 1–9 involving first-row TMs, for which the 3s and 3p orbitals are correlated. The MOBH35/II-III subset contains the remaining reactions involving second- and third-row TM complexes, for which the 3s and 3p orbitals are included in the core region. For both subsets, the percent of canonical CCSD(T) correlation energies recovered by DLPNO-CCSD(T) for various PNO settings and core settings is given in Figure 2.

For the MOBH35/II-III subset (Figure 2a), the correlation energy smoothly converges to the canonical reference by tightening the *T*_CutPNO_ parameter. The results are already reasonably close to convergence with *T*_CutPNO_ = 10^–7^. As expected, CPS(*X*/*Y*) extrapolation provides typically analogous results to those found with *Y* + 1, consistent with previous findings.^21^

In contrast, for the MOBH35/I subset of first-row TM complexes (Figure 2b), the absolute energy error remains large even with extremely conservative *T*_CutPNO_ values. When the tightened semicore settings are used for the MOBH35/I set (see Figure 2c), the error reduces significantly but remains still large and scattered. Interestingly, an overall decrease of wall-clock time was observed with the tightened semicore settings (see Figure 2d). This effect originates from the fact that fewer coupled-cluster iterations are needed to reach convergence when the 3s and 3p orbitals are treated with more conservative *T*_CutPNO_ settings. This efficiency gain increases with the system size.

These results demonstrate that the tightened semicore settings improve both the accuracy and the efficiency of DLPNO-CCSD(T) calculations involving first-row transition metals. Hence, these will become the new defaults starting from the next release of the ORCA package. Unless otherwise specified, these settings are also used in all of the following DLPNO-CCSD(T) calculations.

Despite this significant improvement, the deviation between canonical and DLPNO coupled cluster is still larger for MOBH35/I compared to MOBH35/II-III. As it will be discussed in the next section, this effect originates from correlation effects that are not described by the LMP2 guess used in DLPNO-CCSD(T).

### 3.2 Dynamic Correlation-Induced Orbital Relaxation Effects

In the literature, a diagnostic that is often employed to judge the multireference character of a chemical system is the *T*_1_ parameter,^58,59^ which is defined as the Euclidian norm of the single-substitution amplitude vector (*t*_1_) of the CCSD wave function divided by the square root of the number of correlated electrons (*n*), *i.e*.
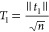
2

This interpretation of *T*_1_ as a measure of the multireference character and thus of possible static correlation effects has been questioned in the past by many authors.^60,61^ Rather than static correlation, the single-excitation amplitudes in coupled-cluster theory describe, to a large extent, dynamic correlation-induced orbital relaxation (DCIOR) effects. This is evident since the operator exp (*T̂*_1_) = exp(∑_*i*,a_*t*_a_^*i*^a^+^*i*) may be viewed as one half of the orbital relaxation operator exp(κ̂) = exp(∑_*i*,a_κ_a_^*i*^(a^+^*i* – *i*^+^a)) and acts in a very similar way. Hence, the dominant effect of *T̂*_1_ is to change the orbitals of the reference determinant according to the dynamic correlation field, which has nothing to do with static correlation effects. We believe that *T*_1_ should be taken as a measure of the adequacy of the reference determinant orbitals (usually HF orbitals). However, quite evidently, this has an impact on the quality of the generated PNOs. If the reference orbitals change strongly in the dynamic correlation field, so would the PNOs that are consistent with the final coupled-cluster wave function. In practice, however, the PNOs are generated by second-order perturbation theory, which most certainly will break down, if the reference orbitals are inadequate. Hence, the larger the *T*_1_–diagnostic is, the less adequate the generated PNOs will be. This issue could potentially be addressed by iterating the PNOs themselves as proposed by Meyer^62^ and was recently also explored by Valeev and co-workers.^63^ However, this comes at the price of highly increased computational cost. Below, we will quantitatively explore the relationship between the *T*_1_–diagnostic and the PNO error for a set of challenging systems in some detail.

**Figure 3:**
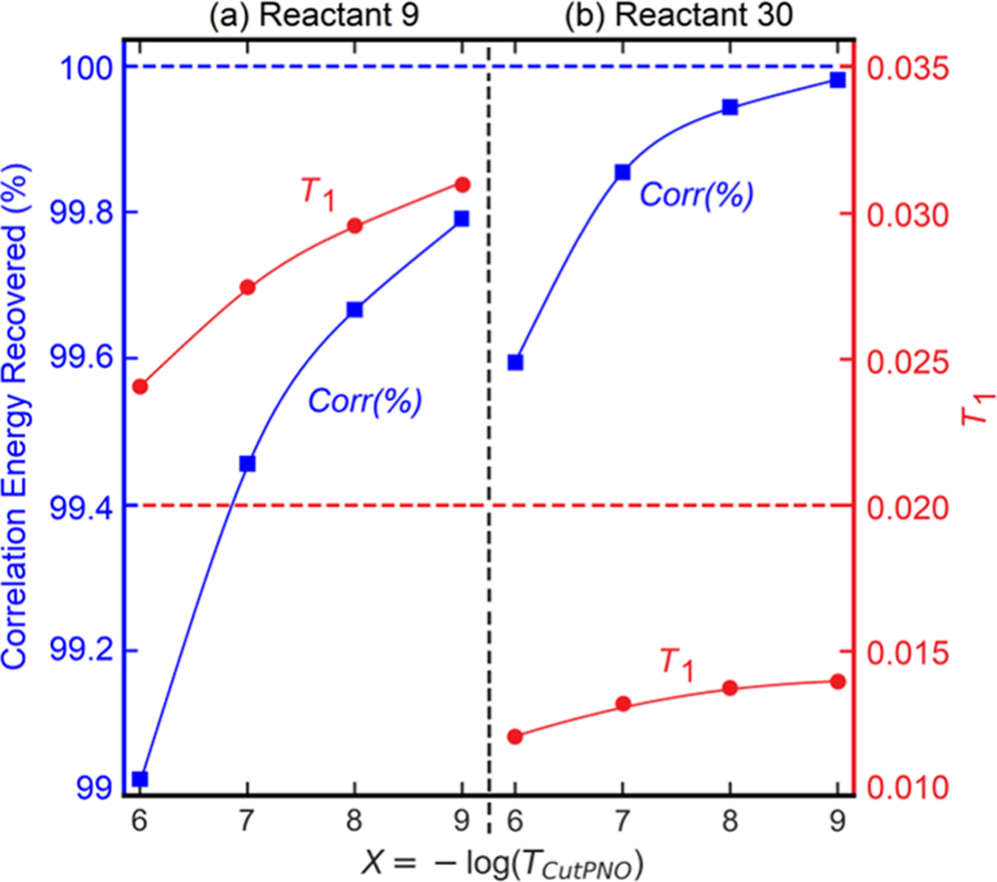
Dependence of the *T*_1_–diagnostic (in red) and the percent recovery of the DLPNO-CCSD(T)/TightPNO/def2-SVP correlation energy (corr (%) in blue) computed with the traditional settings in reference to the CCSD(T)/def2-SVP correlation energy on the size of the PNO space controlled by the *T*_CutPNO_ threshold set to 10^–*X*^ (*X* = 6–9) for reactant complexes (a) 9 and (b) 30 in the MOBH35 set.

Importantly, all molecular systems contained in the MOBH35 set with *T*_1_ values larger than 0.02 show slow convergence with respect to the size of the PNO space (see the ABSOLUTE_ENERGY sheet of the Supporting Information). As an illustrative example of the relationship between *T*_1_–diagnostic and the DLPNO error, the *T*_1_–diagnostic and the percent of canonical CCSD(T) correlation recovered with DLPNO-CCSD(T) are plotted in Figure 3 as a function of the *T*_CutPNO_ parameter for two systems in the MOBH35 set showing drastically different behaviors. Interestingly, the system with larger *T*_1_ is also converging slower toward the canonical limit by tightening *T*_CutPNO_. These results corroborate the already mentioned relationship between DCIOR effects and the DLPNO error and suggest that the *T*_1_–diagnostic can be a useful parameter in this context.

It is worth emphasizing here that, irrespective of the magnitude of *T*_1_, DLPNO-CCSD(T) always converges to the canonical limit when *T*_CutPNO_ is set to 0. Hence, the large PNO truncation error observed in some cases for systems containing first-row TMs originates from the failure of the underlying LMP2 guess in describing DCIOR effects. This in turn causes a deterioration of the quality of the PNOs, which become more delocalized and with a broader eigenvalue distribution. As a consequence, for systems with large orbital relaxation, it becomes necessary including a large number of PNOs to converge toward the canonical limit. These results suggest that the use of more sophisticated wave function-based methods for the initial guess could facilitate the convergence of the DLPNO-CCSD(T) correlation energy toward the canonical limit. Work in this direction is currently in progress in our laboratory.

### 3.3 Dynamic Correlation-Induced Orbital Relaxation Error

Our goal in this section is to provide a diagnostic that could be used by computational chemists to estimate the DLPNO error associated with the PNO truncation in standard DLPNO-CCSD(T) calculations.

**Figure 4:**
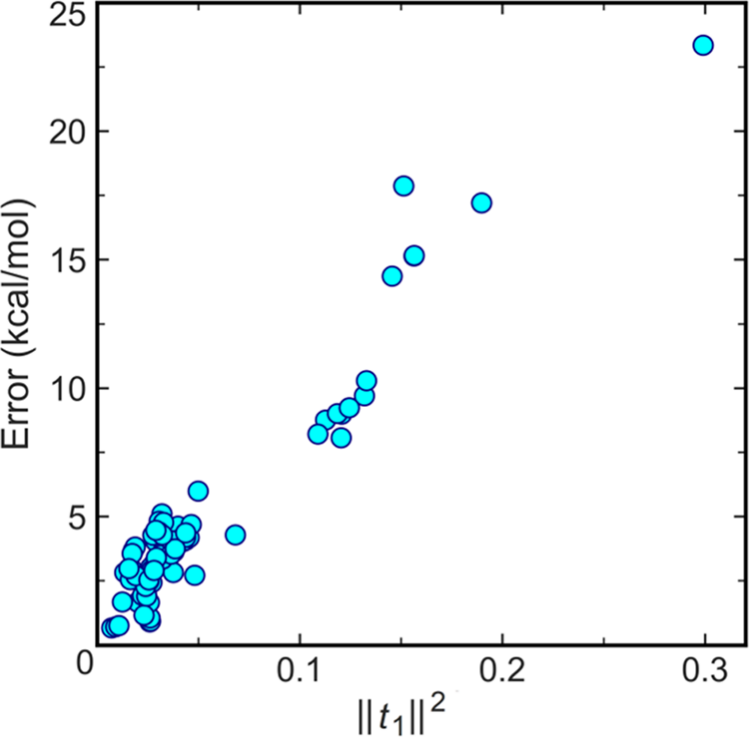
DLPNO-CCSD(T)/TightPNO/def2-SVP (with *T*_CutPNO_ = 10^–7^) error (kcal/mol) for absolute energies of all stationary points of the MOBH35 set as a function of the square of the norm of single-amplitude vector ||*t*_1_||^2^. The results belong to the tightened semicore settings for the first-row TM complexes, while they belong to the traditional settings for the other complexes.

While the *T*_1_–diagnostic can be used for determining potentially problematic systems, it cannot be used to estimate the error quantitatively. The problem stems from the fact that the DLPNO error increases with the system size,^22^ while the *T*_1_–diagnostic is size-independent by definition. Thus, as a semiquantitative diagnostic, we propose instead using the square of the norm of the single-amplitude vector, *i.e.*, ||*t*_1_||^2^, which is a size-consistent quantity. Importantly, for the MOBH35 set, the DLPNO error shows a roughly linear correlation with ||*t*_1_||^2^ irrespective of the *T*_CutPNO_ value (see Figure S1 in the ABSOLUTE_ENERGY_ERROR sheet of the Supporting Information for *X* = 6–9 and also Figure 4).

**Figure 5:**
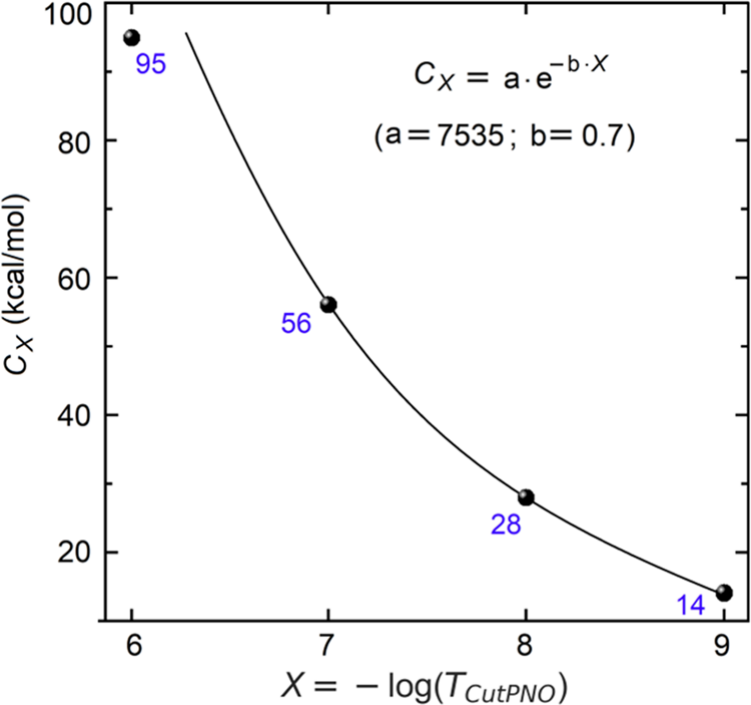
Optimal *C*_*X*_ coefficients (the values in blue) of the linear relation between DLPNO-CCSD(T)/TightPNO/def2-SVP error and ||*t*_1*,X*_||^2^ together with its variation with the size of the PNO space.

The linear relation suggests that it might be possible to estimate the DLPNO error originating from DCIOR effects quantitatively using ||*t*_1_||^2^. We can define the DCIOR energy contribution (Δ) missing in the standard TightPNO energy obtained with *T*_CutPNO_ = 10^–*X*^ as
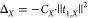
3where *C*_*X*_ is a positive constant and the subscript “*X*” is used to emphasize that all quantities are influenced by the *T*_CutPNO_ parameter. For each *X*, optimal *C*_*X*_ values (kcal/mol) are given in Figure 5 (see the values in blue).

As shown in the “BASIS_SET_DEPENDENCY” sheet of the Supporting Information, Δ_*X*_ is not much sensitive to the basis set type and size, and hence, we suggest using def2-SVP-derived *C*_*X*_ coefficients for estimating the DCIOR contribution with all basis set combinations.

**Figure 6:**
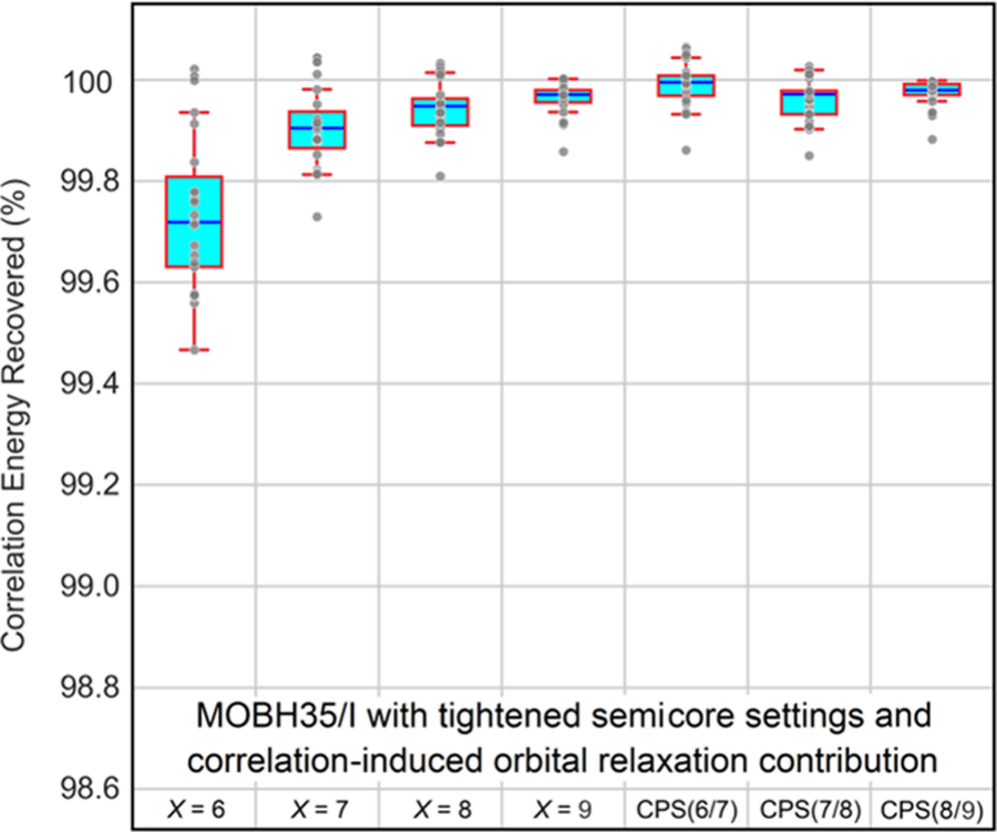
In reference to the CCSD(T)/def2-SVP correlation energies, the percent recovery of the DLPNO-CCSD(T)/TightPNO/def2-SVP absolute correlation energies with varying sizes of PNO spaces controlled by the *T*_CutPNO_ threshold set to 10^–*X*^ (*X* = 6–9) and with different CPS(*X*/*Y*) schemes on all complexes of the MOBH35/I subset using both tightened semicore settings and the correlation-induced orbital relaxation contribution.

**Figure 7:**
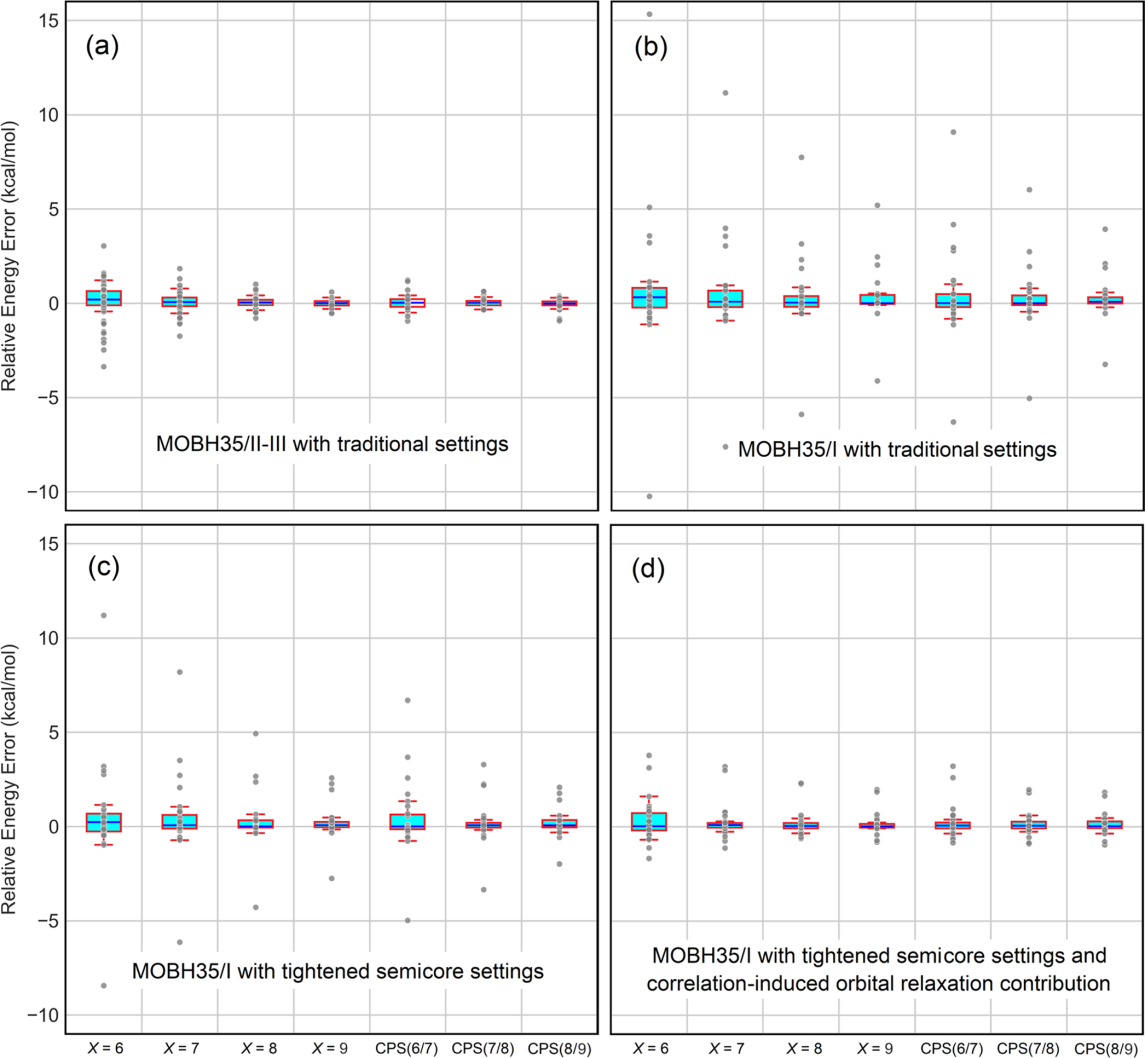
In reference to CCSD(T)/def2-SVP, the error in DLPNO-CCSD(T)/TightPNO/def2-SVP relative energies with varying sizes of PNO spaces controlled by the *T*_CutPNO_ threshold set to 10^–*X*^ (*X* = 6–9) and with different CPS(*X*/*Y*) schemes for (a) the MOBH35/II-III subset with the traditional settings, (b) the MOBH35/I subset with the traditional settings, (c) the MOBH35/I subset with the tightened semicore settings, and (d) the MOBH35/I subset with both tightened semicore settings and the correlation-induced orbital relaxation contribution.

As the DCIOR contribution correlates remarkably well with the DLPNO error, it could in principle be used to correct DLPNO-CCSD(T) energies in those situations for which there is a significant orbital relaxation effect. Indeed, when absolute correlation energies for the MOBH35/I subset are corrected with the DCIOR contribution, the DLPNO error vanishes to a large extent (Figure 6). In particular, CPS(6/7) extrapolation provides correlation energies that are at convergence with respect to the PNO parameter. Importantly, the inclusion of the DCIOR contribution leads to an overall increase in the accuracy of DLPNO-CCSD(T) also for relative energies, as shown in Figure 7. This behavior is also reflected in a decrease of MAEs for relative energies, as shown in Table 1.

**Table 1:** MAEs (kcal/mol) for the DLPNO-CCSD(T)/TightPNO/def2-SVP Barriers and Reaction Energies on the MOBH35 Set with Different PNO Settings in Reference to Canonical CCSD(T)/def2-SVP

	traditional	tightened semicore	tightened semicore + DCIOR
*X* = 6	1.08	0.96	0.60
*X* = 7	0.71	0.62	0.37
*X* = 8	0.47	0.37	0.25
*X* = 9	0.31	0.25	0.18
CPS(6/7)	0.56	0.49	0.35
CPS(7/8)	0.37	0.29	0.22
CPS(8/9)	0.26	0.22	0.18

As a note of caution, it is worth mentioning that small or highly symmetric organometallic complexes typically feature a high degree of d-orbital degeneracy and/or of π-orbital degeneracy associated with the coordinating atom.^58,59,64−67^ In such cases, all single-reference methods, including MP2 and canonical coupled cluster, may fail even qualitatively.^11,58,59,64−67^ In addition, open-shell systems with many unpaired electrons typically feature large *T*_1_ values,^11,59^ which might not necessarily correlate with an increased DLPNO error. For all of these reasons, the DCIOR contribution estimate should not be considered as a generally valid, quantitative energy correction for DLPNO-CCSD(T) calculations. However, it is a useful diagnostic that can be used to determine when a thorough analysis of the convergence of the DLPNO-CCSD(T) correlation energy with respect to the *T*_CutPNO_ parameter is required.

## 4 Conclusions

In this paper, we examined the two primary error sources that affect the accuracy of DLPNO-CCSD(T) energy calculations involving first-row TMs with a complex electronic structure: semicore correlation and orbital relaxation effects. A computational strategy that allowed us to drastically reduce the DLPNO error associated with the 3s3p correlation was presented. In addition, a useful diagnostic for estimating the DLPNO error for systems with high dynamic correlation-induced orbital relaxation was introduced. Finally, our results suggest that improving the quality of the initial guess used in the PNO generation might lead even more robust PNO-based coupled-cluster methods, and efforts in this direction are currently underway in our laboratory.

## Supporting Information Available

Computational details; ORCA input file structure; code for regression analysis, and detailed energetics and error analyses, including *T*_1_–diagnostic (XLSX)
